# A Viral-Human Interactome Based on Structural Motif-Domain Interactions Captures the Human Infectome

**DOI:** 10.1371/journal.pone.0071526

**Published:** 2013-08-08

**Authors:** Aldo Segura-Cabrera, Carlos A. García-Pérez, Xianwu Guo, Mario A. Rodríguez-Pérez

**Affiliations:** 1 Laboratorio de Bioinformática, Centro de Biotecnología Genómica, Instituto Politécnico Nacional, Reynosa, México; 2 Laboratorio de Biomedicina Molecular, Centro de Biotecnología Genómica, Instituto Politécnico Nacional, Reynosa, México; George Mason University, United States of America

## Abstract

Protein interactions between a pathogen and its host are fundamental in the establishment of the pathogen and underline the infection mechanism. In the present work, we developed a single predictive model for building a host-viral interactome based on the identification of structural descriptors from motif-domain interactions of protein complexes deposited in the Protein Data Bank (PDB). The structural descriptors were used for searching, in a database of protein sequences of human and five clinically important viruses; therefore, viral and human proteins sharing a descriptor were predicted as interacting proteins. The analysis of the host-viral interactome allowed to identify a set of new interactions that further explain molecular mechanism associated with viral infections and showed that it was able to capture human proteins already associated to viral infections (human infectome) and non-infectious diseases (human diseasome). The analysis of human proteins targeted by viral proteins in the context of a human interactome showed that their neighbors are enriched in proteins reported with differential expression under infection and disease conditions. It is expected that the findings of this work will contribute to the development of systems biology for infectious diseases, and help guide the rational identification and prioritization of novel drug targets.

## Introduction

The viral proteins hijack cellular machinery by interacting with human proteins. Information from virus-host protein interactions can be employed to predict functions and suggest roles for viral proteins [1], guide experimental strategies for identifying essential host pathways in the viral diseases [2]–[6], and for the discovery of novel drug targets [7]. For example, large-scale studies of virus-human protein interactions, human gene expression under viral infection conditions, and small interference RNA studies to identify human genes that are necessary for virus survival and replication, and computational approaches have been extensively applied for to the human immunodeficiency virus (HIV), the hepatitis C virus (HCV), and the influenza A virus [8]–[10]. However, the overlap between results of different functional genomics studies is small [6], a common characteristic of the results produced by high-throughput technologies during the post-genomic era [11]–[13]. The first two large-scale analyses of the yeast interactome showed an overlapping of about 20% [14]. Three independent siRNA screening to find human cellular factors implicated in HIV replication showed an overlap, between any pair of screens, of less than 7% [8]–[10]. Thus, even though high-confidence data sets are limited, they still provide a framework onto which other types of biological information can be integrated. Recently, several low-throughput studies have shown that interactions among viral and human proteins are mediated by peptide-domain interactions [15]–[19]. For example, the E6 protein of the high-risk mucosal human papilloma virus (HPV) carries the consensus PDZ-binding motif X-T/S-X-V/L at their C-terminus via which the PDZ-containing proteins are targeted. While, low-risk HPV E6 does not present such PDZ-binding motif [20]–[23]. Similarly, the polyproline motif in NS1 protein of influenza A virus contributes to its interaction with p85 regulatory domain of the phosphatidylinositol 3-kinase and induces the activation of associated pathways [24], [25]. It is well-known that motif-domain interactions are mainly involved in signaling networks and transient protein-protein interactions [26]–[30]. Because of their transient nature they are much more difficult to handle in large-scale experiments, thereby, they are poorly represented in related databases [31]. Franzosa and Xia [32] have depicted the structural principles within the human-virus protein-protein interaction network. They reconstructed the human-virus structural interaction network by mapping curated and predicted 3D structural models of human-virus and human-human protein complexes on protein interaction networks from databases. They found that viral proteins tend to interact with human proteins by mimicking and competing for the interaction interfaces of their binding partners within human interactome. Likewise, they showed that viral proteins frequently achieve interface mimicry without any sequence or structural similarity to a human binding partner and the interaction among viral and human proteins are transient and regulatory in nature. Here, we proposed a structural and systems-based approach to predict peptide-domain interactions among viral and human proteins through the identification of structural descriptors from motif-domain interactions of protein complexes deposited in the Protein Data Bank (PDB). The descriptors were used to reconstruct the viral-host interactome for five clinically important human viruses and the implications of the host proteins targeted by viruses in the context of a human interactome were explored. A set of predictions obtained by our method can be used to explain the findings from functional genomics studies documented elsewhere. Our viral-host interactome was able to capture several basic properties of experimental derived host-pathogen interactomes and to further expand the human infectome. In addition, the present work provided a systems-based hypothesis for the understanding of the overlapping between the infectome and diseasome. Therefore, our structure and systems-based approach could complement and guide further high-throughput experiments aimed to identify human genes that are necessary for virus survival and replication in the host cells and will contribute to understanding the mechanism of pathogenesis associated with viral infections, which could prioritize drug targets for a rational antiviral-design.

## Results/Discussion

### Structural Human-viral Interactome Based on Structural Motif-domain Interactions

**Figure 1:**
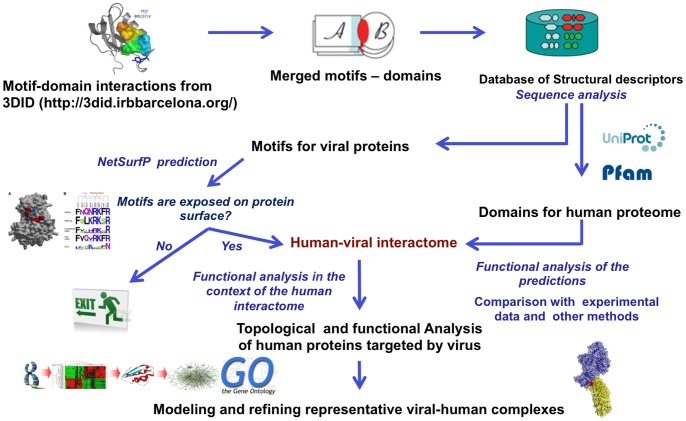
Protocol for the prediction of the viral-human interactome based on structural motif-domain interactions.

**Figure 2:**
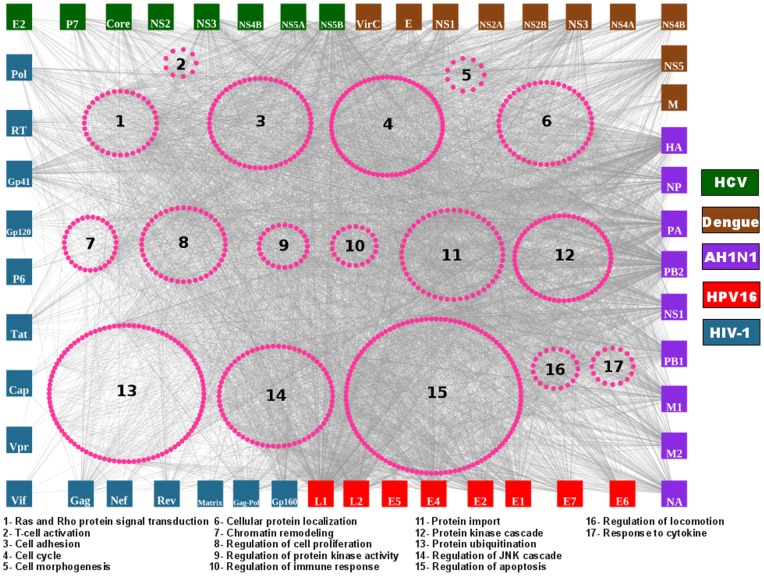
The viral-human interactome for five clinically important viruses. The nodes with squared shape represent to the viral proteins. Nodes with circular shape and magenta color represent the human proteins. The numbers represent to over-represent pathways within the viral-human interactome.

**Figure 3:**
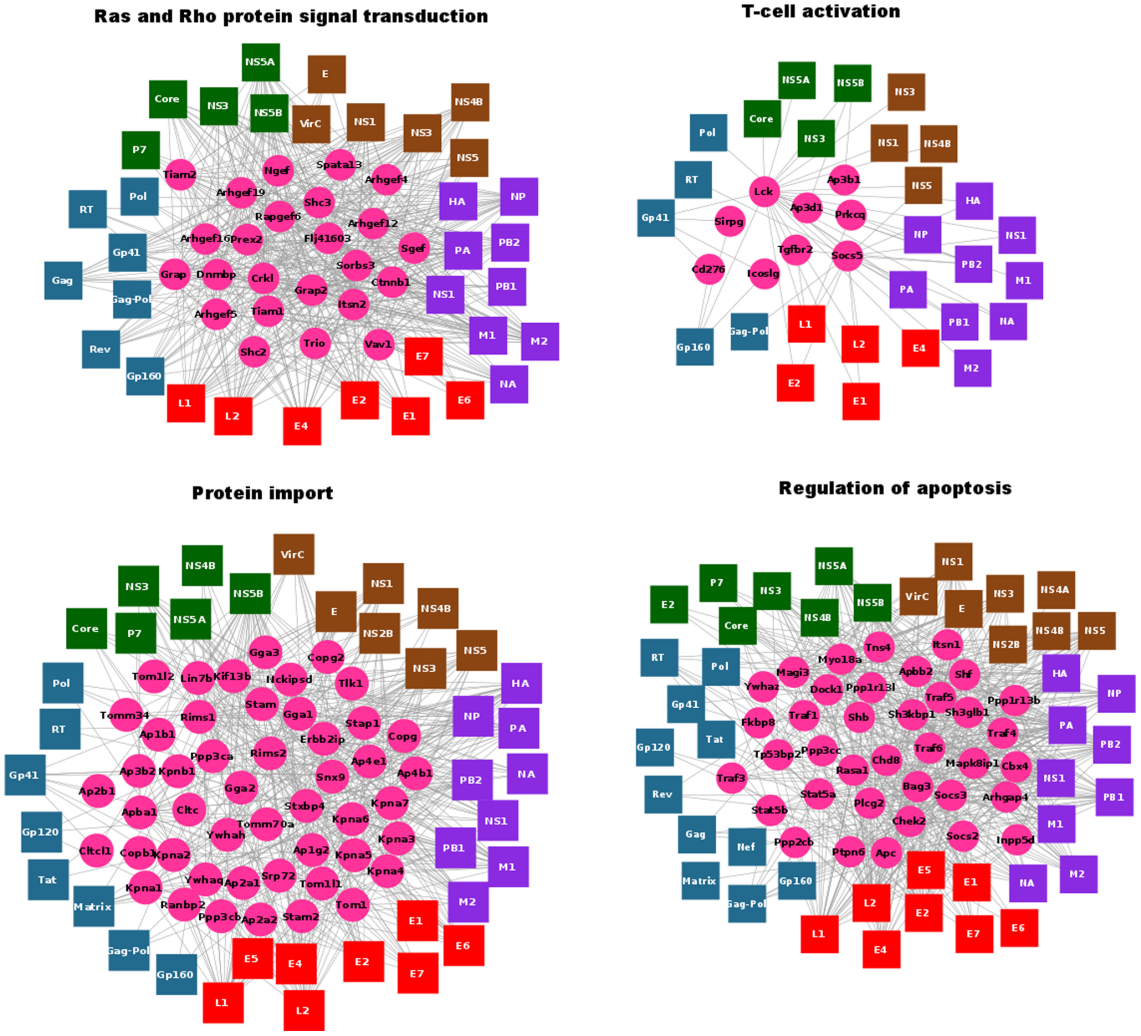
Examples of over-represent pathways targeted by viral proteins.

The human-viral interactome was modeled as an interaction network driven by physical interactions between short peptides (motifs) and domains of viral and human proteins, respectively (Figure 1). The initial interactome was composed of 51 viral proteins of 5 clinically important viruses and 2,094 human proteins throughout 30,196 interactions. Table 1 presents the viruses analyzed in this paper. The motif-mediated interactions occur when a globular domain in one protein recognizes a short linear peptide (motif) from another, creating a relatively small interface of ∼350 Å^2^ on average. The motifs are generally of less than 15 residues in length and defined by a set of sequence, structural and functional attributes. The main caveat of the motif-based predictions is the high rate of false-positives because the motifs are relatively short, thus it is likely that motifs may occur in a protein by random chance. In order to reduce the rate of motif false-positives in the viral sequences a filter using solvent accessibility of motif residues was implemented. Such filter has shown to reduce rate of false-positives as showed elsewhere [33]. Therefore, the final interaction network includes 13,452 interactions (Figure 2 and 3; File S1) between 50 viral proteins (Table S1) and 1,654 human proteins, which represent the ∼7% of the human proteome. Similar results were obtained by Navratil *et al*., when they used a high quality dataset manually curated and validated for virus-host protein interactions to depict the “human infectome” and showed that the 5% of human proteome is targeted by viral proteins [2]. 102 interactions (0.8%) predicted by our approach have been found in VirHostNet database [34], which is higher than true positives found by predictions based on randomly selected viral-human protein pairs (0.07% [average], *P-value* <2.2 10^e-16^). The 102 interactions involved 23 viral proteins and 79 human proteins.

**Table 1:** Viruses analyzed.

Virus	Description	NCBI Taxonomy ID
Influenza	A/Mexico City/001/2009(H1N1))	665622
Dengue	Serotype 2, Strain Thailand/16681/1984	31634
HCV	Hepatitis C virus strain H77	63746
HIV	Human immunodeficiency virus type 1	11676
HPV16	Human papillomavirus type 16	333760

Recently, other approaches for predicting protein-protein interactions between human and viral proteins have been developed; most of them have focused on HIV-1 and human protein interactions [35], [36] and one for dengue virus [37]. Evans *et al.* [35], implemented a protocol based on protein interactions mediated by conserved short eukaryotic linear motifs (ELMs) on multiple sequence alignments of HIV-1 proteins and human protein counter domains (CDs) known to interact with these ELMs. They predicted 109 true positives predictions out of a total of 4,523 predictions. On the other hand, Doolittle and Gomez [36] have predicted interactions between HIV-1 and human proteins based on structural similarity of HIV-1 proteins to human proteins having known interactions. They predicted, after filtering and using the shared Gene Ontology cellular component, 22 true positives predictions out of 265 predictions. In order to compare the results, we analyzed our HIV-1 predictions and those from the above mentioned studies using three reference sets (Table 2): the HIV-1 Human Protein Interaction (NIAID) [38], [39] and the VirHostNet [34] databases, and the HIV-human protein complexes reported by Jager et al., [40].

**Table 2:** Comparison of methods of predicting HIV-human protein-protein interactions.

HIV-human PPIs	Totalpredictions	Match with VirHostNet [34]	Match with HIV NCBI [38], [39]	Match with Jager et al., [40]	Shared predictionsbetween methods
Ours	1,937	11	41	22	
Doolittle and Gomez [36]	265	2	12	23	12
Evans et al. [35]	4,523	21	109	74	331

The comparative analysis of the true positive results found by the three methods did not show significant differences when the VirHostNet and NIAID databases were used (one-tailed exact Fisher test; *P-value* >0.05). The significant differences occurred when Jager et al., dataset was used (one-tailed exact Fisher test; *P-value* <0.05). Such differences could be associated to the methodology used to produce the predictions and the different nature of the reference sets. For example, VirHostNet and NIAID databases integrate literature-curated interactions, and the dataset produced by Jager et al. [40] contains interactions from an affinity binding and purification mass spectrometry (AP-MS) experiment. Doolittle and Gomez [36] used structural similarity information between viral and human proteins to predict interactions. This approach involves complete structural similarity, at protein or domain level, thus their predicted protein-protein interactions should involve large protein interfaces, which are common characteristic in stable proteins interactions [30]. The best match of the results obtained by Doolittle and Gomez method was with AP-MS dataset, which is enriched in stable protein-protein interactions as showed elsewhere by Jager *et al*., [41]. This observation is supported considering that the human proteins present in the AP-MS dataset are enriched in protein domains involved in stable interactions rather than transient interactions (Supplementary Table 4 in Jager et al., [40]). Conversely, the Evans *et al.* and our method utilize information of domain-peptide interactions, which are well-known to be involved in signaling networks and transient protein-protein interactions [26]–[30] and are much more difficult to handle experimentally and, therefore, underrepresented in protein-protein interaction databases [31].

It is worth nothing that the Evans *et al.*, [35] method used only sequence information and associations between motifs and counter domains annotated in Eukaryotic Linear Motif database [42]. Instead, we used peptide-mediated protein interactions of known three-dimensional structure to predict interactions among viral and human proteins followed by a filter including motif surface accessibility. Therefore, Evans *et al.* and our method utilizes in a different manner, information of domain-peptide interactions. This is even more evident when the overlapping between the true positives interactions found by the three methods was analyzed. The results showed only one true positive found by the three methods suggesting that an integrative approach should improve the predictive accuracy of the aforementioned methods. The same applies for the dengue virus. Doolittle and Gomez [37] used a method to predict interactions between the dengue virus and the human host proteins. They predicted 2,073 interactions among viral and human proteins and found 7 out of 19 true positives according their reference dataset. Recently, Khadka *et al.* [43], have identified dengue-human protein interactions by using high-throughput yeast two-hybrid assays. They identified 139 interactions between dengue and human proteins. The overlapping between these studies was of three interactions. While our method predict 2,082 interactions among dengue and human proteins and found an overlapping of six interactions when using the Khadka *et al.* [43] dataset and two interactions when using the reference dataset of Doolittle and Gomez [37], respectively. Despite of the methodological differences between ours, and Doolittle and Gomez methods we found an overlapping of 60 interactions. The small overlap between large-scale experiments is a common characteristic during the post-genomic era [11]–[13].

### Analysis of Predicted Interactions: Inferring Novel Protein-protein Interactions between Dengue Virus and Human

It is worth noting that we found direct and indirect support for some predictions by searching in the literature; such interactions are not yet deposited in the virus-host databases. We focused on some examples of predictions involving dengue virus serotype 2. Dengue has becomes a serious arthropod borne disease causing significant global disease burden in its two clinical forms, namely the classic dengue fever (DF) and the dengue hemorrhagic fever (DHF), the latter as the most severe manifestation of the infection.

**Figure 4:**
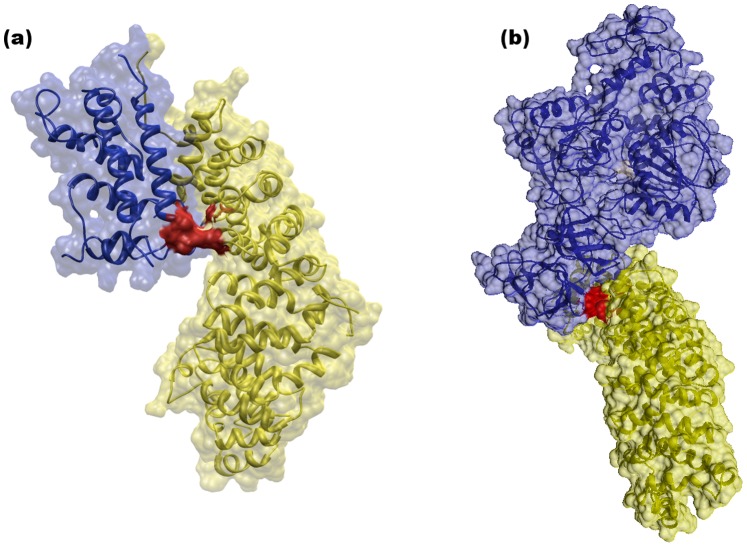
Predicted interaction between VirC protein and alpha subunit from human importin (Kpna1) (a). Predicted interaction between NS3 protein and alpha subunit from human importin (Kpna1). The complexes were modeled using the SwarmDock [51] server for protein-protein docking. The interacting residues are in red color. The structures of VirC and NS3 (both in blue color) and Kpn1 (yellow color) proteins deposited in the PDB, PDB ID: 1R6R, 2VBc and 2JDQ, respectively were used for the protein-protein docking.

It is well-known that VirC protein of dengue virus could be found in the cytoplasm and nucleus of many infected cell lines [44]–[46]; however, its role into the nucleus of human cells is not completely understood. We predict an interaction among VirC protein of dengue virus and human importin subunit alpha-1 (Kpna1). Kpna1 mediates the nuclear import of HIV-1 Vpr, ebolavirus Vp24, influenza virus polymerase (PB2), and human cytomegalovirus UL84 proteins by recognizing a non-classical nuclear localization signal (NLS) [19], [47]–[49]. We identified two motifs on the sequence of VirC that could be potential interaction sites for the Arm domain of human Kpna1 (Table 3). Ma *et al.* [50], have determined the 3D-structure of VirC protein of dengue virus suggesting a model where the group of helices in N-terminal region (α2–α2′) are involved in membrane association and the helices in the C-terminal region (α4–α4′) mediate RNA interactions. The analysis of 3D-structure of VirC showed that one NLS motif is placed at the core of the helices in N-terminal region (α2–α2′) while the other belongs to the loop placed at the end of the helices in the C-terminal region (α4–α4′) without apparent structural role. Sangiambut *et al.* [15], have identified those regions in VirC protein of dengue serotype 2 virus implicating its nuclear localization during the infection process. They introduced double alanine-substitution mutations in the into three NLS motifs at positions 6–9, 73–76, 85–100 and found that mutations in K73A, K74A and R85A, K86A resulted in an elimination of nuclear localization in PS cells and marked reduction in Vero cells. Since the association between the NLS motifs at positions 73–76, 85–100 and nuclear localization of VirC, they suggested that the protein should be transported into the nucleus via an active process involving proteins within the importin superfamily. The mutations in K73A and K74A are placed in the NLS motif that we predicted interacts with Arm domain from Kpna1 protein. In order to visualize this prediction, a protein-protein docking procedure between the Kpna1 (PDB: 2JDQ) and VirC (PDB: 1R6R) proteins using SwarmDock server [51] was performed. The results showed that the NLS motif at position 73–76 is placed in the NLS-binding site of Arm domain from Kpna1 protein (Figure 4a) in a similar manner as that of NLS motif at the C-terminal region of PB2 domain [19] from influenza virus polymerase. Similarly, we predicted interactions of Kpna1 with NS5 and NS3 dengue proteins. Pryor et al., [52] have demonstrated that nuclear localization of dengue virus NS5 is important for the viral replication and suggest that the NLS motifs in NS5 are recognized by importin α/β as the key signal for the nuclear import. They have mutated a set NLS motifs identified previously in two regions (A and B) of dengue NS5 [53], [54] and found that mutations in the NLS motifs from A region decrease its nuclear localization and the viral replication. In present study, we found an additional NLS motif located at C-terminal region of NS5 (Table 3). Recently, Kumar et al. [55] have systematically mutated all charged amino acids within A region of dengue NS5 and found that the charged amino acids at the C-terminal region are also involved in the nuclear transport of dengue NS5. The NS5-Kpna1 complex has not been modeled yet because the motif at C-terminal region of NS5 was missed in the 3D structures available to date. In the case of NS3-Kpna1 prediction, we also found two NLS motifs (Table 3) as potential biding sites of Arm domain of Kpna1, suggesting a possible nuclear localization of dengue NS3 mediated by this human protein. The predicted complex by docking (Figure 4b) showed an interaction between the NLS motif of NS3 protease domain and key residues of the Arm domain of Kpna1 involved in the import process [19]. Uchil et al. [56], have demonstrated that dengue NS3 is present in the nucleus of its host cells. They found that NS3 co-localizing with NS5 in the host nucleus, suggesting that flaviviral replication is not only restricted to cytoplasm of the host cell but also occurs in host nucleus. Therefore, we provided two NLS motifs with potential implication in nuclear localization of dengue NS3, but further mutagenesis studies to validate this prediction are needed.

**Table 3:** Predictions of interacting motifs on the amino acid sequence of VirC, NS5, NS3 and E proteins of the dengue virus serotype 2.

Dengue protein	Motif positions*	Motif residues	Surface accessibility
VirC	6–9	KKAR	EEEE
	73–76	KKSK	EEEE
NS5	457–460	KREK	EEEE
NS3	142–145	KKGK	EEEE
	589–592	KKLK	EEBE
E	328–333	GDGSPC	EEEEEB
	360–365	EKDSPV	EEEEEB

* Numbering scheme for positions is according to the structures of VirC, NS3 and E proteins deposited in the PDB, PDB ID: 1R6R, 2VBC and 3UAJ, respectively. The numbering scheme used for the NS5 protein was according to the reported by Kumar et al., [55]. E: exposed residue, and B: buried residue.

Taken together, the experimental evidence above-mentioned support these blind predictions of our method.

In addition, we found other interesting interactions with indirect evidence involving VirC of dengue virus and human proteins. These predictions are relevant considering that involve human proteins that are known to be targeted by proteins of several viruses which is consistent with previous observations suggesting that many viruses share common infection strategies [2], [3], [57]. For example, we predicted the interaction between the motif at N-terminal region of VirC and SIR2 domain of the Sirt1 protein. The docking procedure for the prediction of VirC-Sirt1 complex was not performed because of the motif at N-terminal region of VirC was not available in its 3D structure (PDB: 1R6R). Sirt1 is a NAD-dependent deacetylase involved in the transcriptional regulation of biological processes such as cell cycle, modulation of chromatin function by deacetylation of histones, response to DNA damage, apoptosis and autophagy. Pagans *et al.* [17], have shown that Tat HIV-1 protein interacts with Sirt1 and it is mediated by the interaction with a basic charged motif (KKRR) at 50–53 positions on Tat sequence and the SIR2 domain of the Sirt1 protein. Thus, we proposed the following model: Once VirC has gained access to the nucleus; it interacts with Sirt1 inhibiting its deacetylase activity and affecting the desacetylation patterns on their substrates such as H3 and H4 histones. Moreover, Bosh *et al.* [58], have found a relationship between the hyperacetylation of histones H3 and H4 and high expression of IL-8 in human monocytes infected by the dengue virus serotype 2. IL-8 has been found at higher levels in DHF patients than in those patients with DF [59]. Kwon *et al.* [60], found that Tat HIV-1 protein inhibits the deacetylase activity of Sirt1 protein and induces T-cell hyperactivation. The hyperactivation of memory CD8+ T cells during a heterologous secondary dengue virus infection has been suggested to result in a massive production of cytokines (cytokine storm) and immune modulators characteristic of DHF [61]. It appears that VirC of dengue virus play a key role in the so-called cytokine storm by modifying the epigenetic mechanisms associated with the overexpression of cytokine genes. We also predict interactions between VirC and members of canonical Wnt signaling pathway (Catenin beta-1, Catenin delta-1, and Catenin delta-2 proteins). Wnt signaling pathway is involved in regulating key physical and pathological processes including cellular proliferation, differentiation, and transformation [62]. Liu *et al.* [63], have demonstrated that the core protein of hepatitis C virus (HCV), an analog protein to dengue VirC, activates the Wnt signaling pathway and suggested that the core protein may be directly involved in liver pathogenesis. Limjindaporn *et al.* [64], have showed that VirC is involved in apoptosis of virus-infected liver cells. In dengue virus, the Wnt pathway has been associated only to the infection response in *Aedes aegypti* [65], which is a primary vector of the dengue virus worldwide. Even though the role of Wnt signaling pathway in dengue disease is not clear, we provide a set of predictions for further functional genomics studies; moreover, considering that this pathway is preferentially targeted by several viruses such as HCV, Epstein-Barr virus (EBV), and HPV-16 as shown elsewhere [66]–[69].

**Figure 5:**
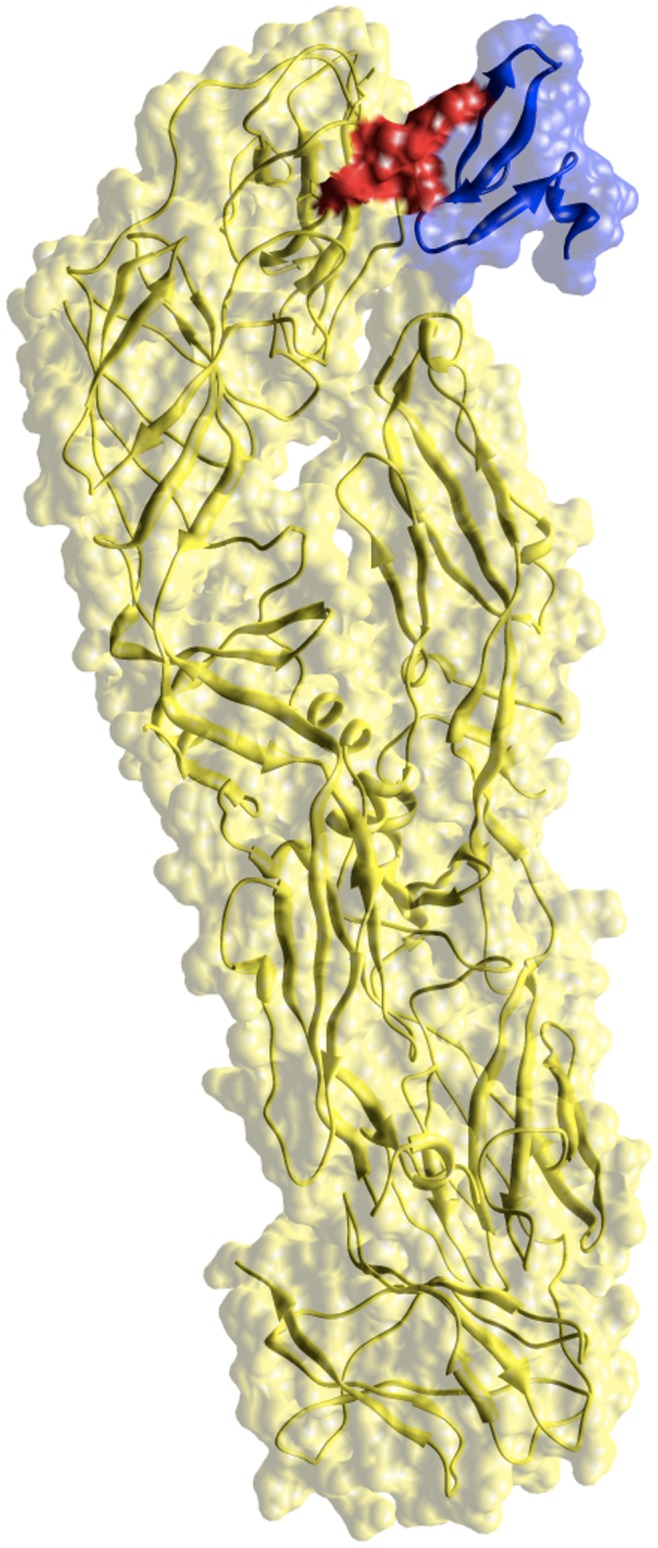
Predicted interaction between E protein and WW domain (red color). The complex was modeled using the SwarmDock server for protein-protein docking. The structures of E protein (blue color) and WW domain (yellow color) deposited in the PDB, PDB ID: 1TG8 and 2KQ0, respectively were used for the protein-protein docking.

Likewise, we predicted an interaction between the enveloped protein of dengue virus (E) and the Itch human protein (Figure 5). Itch belongs to a group of protein-ubiqutin ligases of the Nedd4 family. This family of proteins is targeted by several viral proteins such as Epstein-Barr virus LMP2A protein, HPV-16 E6 protein, and HTLV-1 Gag protein from which interactions are mediated by the WW-binding motif of viral proteins and the WW domain of human proteins. We identified two WW-binding motifs at C-terminal region of the E protein, which are placed at domain III, an immunoglobulin (Ig)-like domain that is thought to contain potential biding-sites (Table 3). Itch protein is involved in the regulation of innate immune response by degrading the MAVS protein and preventing the harmful effects of an exacerbated immune response on the host. You *et al.* [70], showed an exaggerated and prolonged antiviral response in Itch deficient mouse embryonic fibroblasts. Thus, E protein could be affecting the regulation of innate immune response by blocking the degradation of MAVS protein, which is associated to an exacerbated immune response on the host, being a common pattern in severe dengue infection.

### Analysis of Human Proteins Targeted by Virus in the Context of Human Interactome

The viral-host interactome present here is composed of 50 viral proteins and 1,654 human proteins (predicted as human viral targets). We found that 79 human proteins from the 1,654 above mentioned have interactions with viral proteins deposited in the VirHostNet database. Functional analysis of 79 human proteins showed that they are associated to biological processes implicated in viral infections. The remaining 1,574 human proteins predicted to interact with viral proteins were analyzed by functional enrichment analysis in order to find shared biological processes with the set of 79 targeted human proteins. The analysis showed that 1,224 proteins shared biological processes annotations with the 79 human proteins targeted by the virus (Figure 2 and 3).

**Figure 6:**
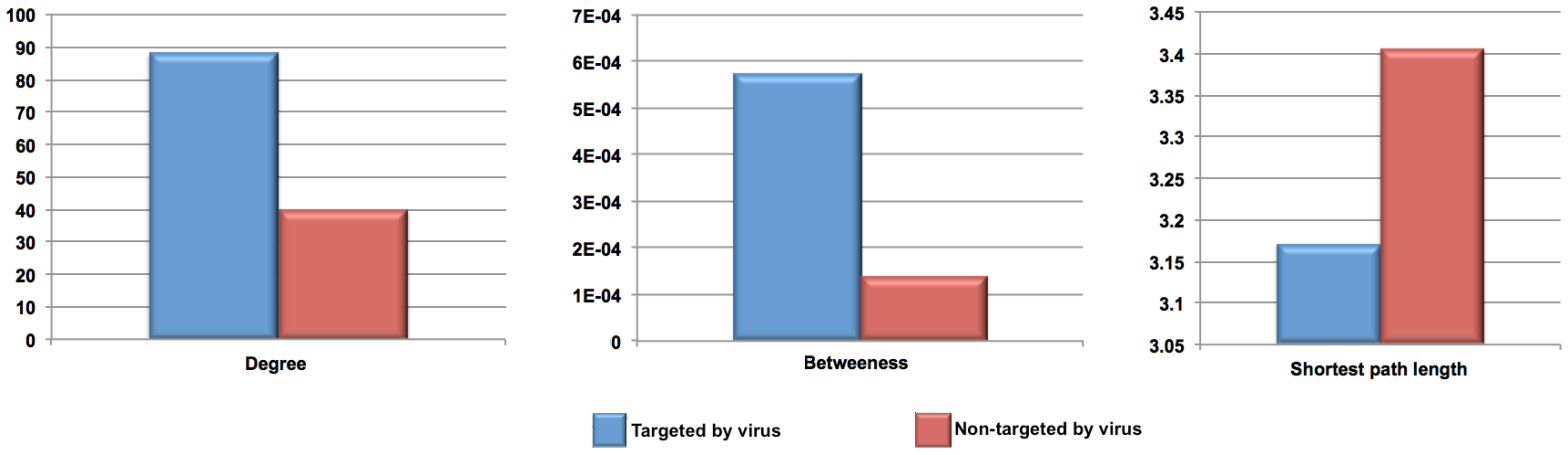
Analysis of the topological properties of the human proteins targeted and non-targeted by virus, respectively, in the context of a human interactome. The average degree, betweenness and shortest path length properties of human proteins targeted by virus (blue bars) are compared to that of human proteins non-targeted by virus (red bars).

The analysis of the predicted human viral targets in the context of functional genomics data showed that the 26% (430) of them have been found with differential expression in humans under several infection conditions in at least two experiments deposited in the Gene Expression Atlas. Additionally, the ∼30% (487) of the predicted human viral targets have been reported previously interacting with other human pathogens. Moreover, these proportions share the ∼35% (164) of the predicted human viral targets. Thus, the analysis of the predicted human targets was able to associate the 46% (430+487–164 = 753) of predicted human viral targets to the proteins that were identified to be related to pathogen infections obtained from functional genomics. Hence, although our method shows poor overlapping with experimental viral-host interactions, it is still able to capture a large proportion of the proteins that were identified to be related with pathogenic infections from functional genomics data. This proportion of human viral targets is consistent with several experimental reports. For example, Khadka et al., 2011, performed a similar analysis of their dengue-human interactome and found that the 43% (45 out of 105) human dengue targets have overlapping with functional genomics data associated with pathogeńs responses. The statistical analysis of proportions mentioned previously were significant in comparison to the average values of 100,000 random lists of equal size generated from reference human proteome (one-tailed exact Fisher test; *P-value* <2.2 10^e-16^ and *P-value* <1.3 10^e-10^, respectively). Therefore, our findings are consistent with the current evidence that suggest a common viral infection strategy could exist since the proteins from different virus attack the same key proteins within the human interactome [2], [3], [57], [71]. Dyer *et al.* [3], have analyzed the landscape of human proteins interacting with pathogens. They integrated human-pathogen PINs for 190 pathogen strains from seven public databases and found that both viral and bacterial pathogens tend to interact with proteins with many interacting partners (hubs) and those that are bottleneck proteins in the human interactome [3]. Similar results were obtained by Navratil *et al.* [2], they used a high quality dataset manually curated and validated of virus-host protein interactions to depict the human infectome. They showed, by using functional genomic RNAi data, that the high centrality of targeted human proteins was correlated to their essentiality for viruses’ life cycle and by topological analysis of the network suggesting a preferential viral attack against central and bridging proteins. Thus, in order to determine if the host-viral interactome presented in here is able to capture the relationship between centrality of human protein and their essentiality for viruses’ life cycle, an analysis of the topological properties of human targeted proteins compared with non-target proteins within a human interactome was performed. The analysis showed significant trends (one-tailed Wilcoxon test; *P-value* <2.2 10^e-16^) of the viral proteins to preferentially interact with proteins that have high degree (connectivity), high betweenness centrality and short path length values compared with those of non-targeted by viral proteins in human interactome (Figure 6) as showed elsewhere [1]–[3]. Besides, a strong association between the connectivity of human proteins within human interactome and its likelihood of being targeted by viral proteins was found (0.98; *P-value* <2.2 10^e-16^). When taken the results together, they support the hypothesis that virus preferentially attacks hubs and bottleneck proteins within the human interactome suggesting that viral proteins have evolved to hijack essential components and pathways of the cells [72]. Likewise, the analysis of the functional context of targeted human proteins showed that the ∼40% (3,198) of their interacting partners have differential expression under several infection conditions, which represents the 70% (3198/4531) of proteins that were able to map within the human interactome. Therefore, we identified that the 31% of the human proteome (1,654+3,198 proteins) is involved in the infectious diseases process.

### Analysis of Human Infectome in the Context of Phenotype Data

To decipher if the human infectome described here is enriched with phenotype data associated to diseases or essential processes, a phenologs analysis was performed. According to McGary *et al.* [73], phenologs are equivalent phenotypes defined by the orthology relationship of the associated genes in two organisms. Using the phenologs approach, they predicted unique genes associated with diseases and suggested several models for disease; a yeast model for angiogenesis defects, a worm model for breast cancer, mouse models of autism, and a plant model for the neural crest defects associated with Waardenburg syndrome [73]. The results revealed that human infectome was enriched significantly by essential and disease phenotypes in mice, worms, yeasts, and plants (Table 4) as compared to the remaining human proteome counterpart (Table 5). Surprisingly, we found 45 orthologs of human infectome in *Arabidopsis thaliana* with a phenotype associated to the defense response to pathogens. For example, the RD19 and AT4G13350 proteins are involved in the resistance to *Ralstonia solanacearum* and in modulating nucleocytoplasmic shuttling of geminivirus nuclear shuttle protein, respectively [74], [75]. While their counterparts in humans correspond to the cathepsin F and Agfg1 proteins which are involved in tumor invasion [76] and HIV-1 replication [77], [78], respectively. Furthermore, the analysis of the 45 orthologs of *Arabidopsis thaliana* in the human infectome showed that 22 of them are associated to phenotype disease such as several types of cancer (lung, liver, colorectal, melanoma, and prostate), porphyria, defective bone mineralization, and Parkinson disease (Table 6).

**Table 4:** Phenologs of human proteins targeted by virus.

Organism	Phenotype	Orthologs	Shared Orthologs	Hypergeometric probability
Mouse	Lethality-prenatal/perinatal	1051	508	5.40e-58
Mouse	Immune system phenotype	883	420	2.49e-45
Mouse	Hematopoietic system phenotype	595	306	3.46e-41
Mouse	Cardiovascular system phenotype	587	301	3.43e-40
Mouse	Tumorigenesis	227	133	2.67e-25
Worm	Locomotion_abnormal	447	256	9.53e-13
Worm	Embryonic_lethal	1174	568	1.53e-08
Worm	Larval_lethal	285	163	2.31e-08
Worm	Maternal_sterile	494	255	8.66e-07
Worm	Organism_morphology_abnormal	155	91	7.80e-06
Yeast	Sensitivity at 5 generations in 10 uM nystatin	86	60	2.03e-05
Yeast	Reduced fitness in rich medium (YPD)	41	32	6.04e-05
Yeast	Sensitivity at 15 generations in 10 uM nystatin	217	130	9.72e-05
Yeast	Rate of growth sensitivity in 0.85 M NaCl	164	100	2.67e-04
Yeast	Growth defect on a non-fermentable carbon source	18	16	3.19e-04
Arabidopsis	Response to cadmium ion	142	95	2.90e-11
Arabidopsis	Protein amino acid phosphorylation	51	42	4.74e-10
Arabidopsis	Regulation of cell cycle	16	16	3.93e-07
Arabidopsis	Response to salt stress	101	57	4.81e-04
Arabidopsis	Response to bacterium	50	30	3.03e-03
Arabidopsis	Response to virus	9	7	2.46e-02

**Table 5:** Phenologs of human proteins non-targeted by virus.

Organism	Phenotype	Orthologs	Shared Orthologs	Hypergeometric probability
Mouse	Homeostasis/metabolism phenotype	829	506	7.31e-08
Mouse	Behavior/neurological phenotype	796	473	1.36e-05
Mouse	Increased circulating thyroid-stimulating hormone level	13	13	2.10e-04
Mouse	Polyphagia	34	28	2.46e-04
Mouse	Endocrine/exocrine gland phenotype	494	294	4.91e-04
Worm	Thin	56	43	2.61e-02
Worm	Mitochondrial_metabolism_abnormal	7	7	4.28e-02
Yeast	Adaptation time sensitivity in 0.85 M NaCl	392	282	4.56e-04
Yeast	Reduced sporulation	17	17	5.83e-04
Yeast	Growth defect on a fermentable carbon source	193	144	1.29e-03
Yeast	Exhibits sensitivity at 5 generations whengrown in synthetic complete medium	149	112	2.97e-03
Arabidopsis	Cell redox homeostasis	11	11	8.30e-03
Arabidopsis	Electron transport	40	33	1.10e-02
Arabidopsis	Vegetative to reproductive phase transition	10	10	1.28e-02
Arabidopsis	Response to red light	10	10	1.28e-02
Arabidopsis	Small GTPase mediated signal transduction	14	13	1.94e-02

**Table 6:** Comparison of Arabidopsis and human phenologs associated to pathogen responses and human phenotype, respectively.

Arabidopsis	Phenotype	Human	Phenotype
AT5G57220	Response to bacteria, fungus,and insects	1543, 1544, 1545, 1548, 1549,1553, 1555	Cancer: lung, liver, colorectal, prostate
AT4G31500	Response to bacteria	1557, 1558, 1559, 1562, 1565,1571, 1572, 1573	Cancer: lung, liver, colorectal, prostate
AT5G57220	Response to bacteria, fungus,and insects	1586, 1589	Cancer: liver, and colorectal
AT5G08280	Response to bacteria	3145	Porphyria
AT5G50850	Response to bacteria	5162	Pyruvate carboxylase deficiency
AT2G30770	Response to bacteria, fungus,and insects	29785	Extrahepatic xenobiotic metabolism, and melanoma
AT2G30770	Response to bacteria,and fungus	120227	Asthma, and defective bone mineralization
AT1G73080	Response to bacteria,and fungus	120892	Parkinson disease

These findings have multiple implications; a set of proteins are conserved across very distantly organisms and they are involved in the response to pathogen infections, thus the cell is recycling proteins and modules to produce different phenotypes as showed by McGary *et al* [73]. Even though the homology relationship between humans and other organisms is not so high or perhaps non-obvious, opens up the opportunity to use them as models in order to improve our understanding of human-pathogen relationships and for other essential processes. For example, Cha *et al.* [79], have identified a group of proteins whose function in yeast is to maintain cell walls while their phenologs have been found to have an alternative use in vertebrates regulating angiogenesis. They reasoned that drugs that modulated the yeast phenologs might also modulate angiogenesis in humans and in animal models counterparts, and found that thiabendazole is indeed able to act as a vascular disrupting agent and decreased vascular density in human tumors grafted into mice. Therefore, the remarkable growth of structural and functional data from human-pathogen systems should enable the analysis of host-pathogen relationships in different models and distant organisms and the application of this to other fields such as in agriculture. Moreover, it suggests a relationship between the pathways associated to pathogen infection and non-infectious diseases (diseasome). This debatable and controversial observation is not new as shown elsewhere by Navratil *et al.* [2], and Gulbahce *et al* [5]. Navratil *et al.* showed that 152 of 1,148 of targeted proteins in the human infectome are significantly and directly associated to a least one disease suggesting that a human protein that directly interacts with a viral protein has roughly two times more chances to be involved in a human disease than those human proteins that are not targeted by a virus. They identified 34 diseases significantly linked to viruses, the majority of them being related to cancers and neurodegenerative diseases, and provided the platform for mapping the molecular associations between viral infection and human diseases based on virus-host physical protein-protein interactions. Gulbahce *et al.* [5], examined how viral perturbations over host interactome may underline the relationships between human diseases and viral infections. They used Epstein-Barr virus (EBV) and human papillomavirus (HPV) as models, and found that host targets of viral proteins reside in network proximity to products of disease susceptibility genes. Likewise, they uncovered a novel pathway linking HPV to Fanconi anemia. These results support the conclusion that the diseasome and infectome are connected. Further, we reasoned that infectome belongs to diseasome and that unexpected overlapping between viral targeted and non-infectious diseases proteins/pathways could be explained considering that such proteins/pathways are the basic elements of diseasome that need to be perturbed allowing the disease to arise. As a consequence, the final phenotype disease arises as emergent property from interactions with other human and pathogen proteins, environmental factors or drugs. This structure and systems-based approach must complement and guide further high-throughput experiments aimed to identify human genes that are necessary for virus survival and replication, and contribute towards the understanding of the pathogenesis mechanism associated with viral infections. Consequently, to prioritize drug targets for a rational antiviral-design.

In conclusion, we built a single viral-human protein-protein interaction network based on motif-domain structural descriptors. This allowed identifying and prioritizing a set of new protein interactions that further explains the molecular mechanism associated with viral infections. Our results suggest that targeting the nuclear trafficking pathway of viral proteins is an attractive target for the design of drugs against dengue virus, as proposed by Wagstaff et al. [80], and Caly et al., [81].

The analysis of the viral-host interactome showed that it is able to capture the human infectome. The systems-level analysis of human proteins targeted by viral proteins in the context of human interactome showed that they corresponds to proteins with high connectivity within the human interactome and it is consistent with previous findings suggesting a common viral infection strategy by attacking key nodes of the human interactome; moreover, its neighbors are enriched in proteins and have been reported with differential expression under distinct infection conditions. We proposed a hypothesis to explain the relationship between the human infectome and the diseasome where proteins/pathways targeted by virus or diseases are considered as a hot spots needed to be perturbed allowing the disease arise, and where the final phenotype disease is produced as emergent property from interactions with other elements from the system, namely human proteins, pathogen proteins, and toxic chemicals in the environment or drugs. On the light of the present results, we provided a set of predicted protein-protein interactions between viral and human proteins that can be used in functional genomics studies to identify host factors required for viral infections or through mutagenesis studies of viral proteins in order to identify molecular mechanism associated with viral infection, and better strategies for drug discovery.

## Materials and Methods

The method used protein structural data that describes a motif-domain interaction to build a set of structural descriptors. These are then used to search for human and viral protein sequences.

### Peptide-domain Interactions Gathered from the Protein Complexes Deposited in the PDB

For peptide-domain interactions, data were taken from the 3DID database [82], [83]. 3DID is a collection of domain-domain and motif-domain of proteins for which high-resolution three-dimensional structures are known.

### Motif-domain Structural Descriptors for the Viral-host Interactome

The SwissPfam dataset from Uniprot [84]–[86] database was used to extract domain content of the human proteome sequences (human domain set). The human domain set was used to filter the 3DID database in order to obtain motif-domain interactions involving only domains in the human proteome. The result was a descriptor database, including for each domain in the human proteome its motif partner as regular expression. The resulting information was used for searching motifs on the sequences of viral proteins and linking them to proteins containing its partner domain. Therefore, viral and human proteins, sharing a motif-domain descriptor, were predicted as interacting proteins. The set of viral protein sequences corresponds to the dengue virus serotype 2, HCV, HIV-1, A H1N1 influenza, and HPV16 viruses, which are viruses clinically well-defined. The main caveat of the motif-based predictions is the high rate of false-positives because the motifs are relatively short, thus it is likely that a high rate of motifs would occur in a protein by random chance. In order to reduce the rate of motif false-positives in the viral sequences, a filter using surface accessibility of motif residues was implemented. The surface accessibility of motif residues on viral proteins was predicted using the Netsurfp package [87]. Thus, if more than half of residues from one motif are exposed, it is maintained in the descriptor database, otherwise it is removed. Such filter has shown to reduce the rate of false-positives as documented elsewhere [33]. The performance evaluation was carried out by comparison of our predicted set and the experimental dataset based on the overlap between the two sets. We generated 100,000 random networks with the same sizes as our predicted viral-host interactome in order to evaluate if the overlap between the two sets was statistically significant. The reference dataset corresponds to information extracted from the VirHostNet database [34], which is a high quality dataset manually curated and validated of the virus-host protein interactions. *P*-values for the overlap between prediction and reference datasets were calculated using the R Project for Statistical Computing.

### Analysis of Human Proteins Targeted by Virus in the Context of Human Interactome

In order to evaluate how the viral proteins affect the functioning and the robustness of the cellular network, the topological properties of human targeted proteins were analyzed and compared with non-target proteins within a human interactome. The human interactome was obtained from the InterologFinder resource (http://interologfinder.org). This interactome integrated experimental protein-protein interactions from representative databases, and predicted interactions by interologs method under high confidence scoring scheme [88]. The connectivity (k) of a human protein in the human interactome corresponds to the number of direct interacting partners. The number of partners of a targeted human protein is denoted by node degree value. The betweenness centrality represents the number of shortest paths that pass through a given node. In protein-protein interaction network, the betweenness centrality of a protein is used to estimate the theoretical flux of information that is potentially controlled by this protein and as an unbiased measure of functional importance to network more than just connectivity. The topological properties of the human interactome aforementioned were calculated with igraph package implemented in the R Project for Statistical Computing [89].

The statistical analysis of the differences observed in medians of calculated topological properties was performed by Wilcoxon rank-sum test. The Exact Fisher test was used to test the differences observed between proportions and to test over-representation and functional enrichment.

### Functional Analysis of Human Proteins Targeted by Virus

The Gene Expression Atlas resource was used to obtain information about the expression of human proteins targeted by the virus. The parameters used for searching were: 1) Human; 2) Reported by more than two publications; and 3) Under distinct infection conditions.

The phenologs server (http://www.phenologs.org) was used to identify if human proteins targeted by the virus are enriched with phenotype data associated to diseases or an essential process.

## Supporting Information

File S1 **Tab delimitated file of the viral-human interactome predicted in this paper.**
(TXT)

Table S1 Viral proteins analyzed in the present study. The functional descriptions of viral proteins were collected from NCBI and Uniprot protein databases in May 2013.
(XLS)
